# Relative SNR Measurements in Supine vs. Prone Breast MRI

**DOI:** 10.1002/mrm.70217

**Published:** 2025-12-25

**Authors:** Jeremiah J. Hess, Catherine J. Moran, Preya Shah, Jana Vincent, Fraser J. L. Robb, Bruce L. Daniel, Brian A. Hargreaves

**Affiliations:** ^1^ Department of Radiology Stanford University Stanford California USA; ^2^ Department of Bioengineering Stanford University Stanford California USA; ^3^ Department of Radiology Ohio State University Columbus Ohio USA; ^4^ GE Healthcare Chicago Ohio USA; ^5^ Department of Electrical Engineering Stanford University Stanford California USA

**Keywords:** breast MRI, SNR, supine

## Abstract

**Purpose:**

Supine breast MRI has the potential to improve patient comfort compared to prone breast MRI, in addition to providing images in the same position as subsequent treatment protocols. Novel flexible coil arrays have enabled high SNR and parallel imaging in supine breast imaging, but the combined effect of coil and patient positioning on SNR has yet to be investigated. The aim of this study is to use a tissue‐independent metric to account for tissue deformation to compare SNR between prone and supine positions, using appropriate coils for each.

**Methods:**

Relative SNR (rSNR) metric is proposed as the ratio of SNR between a breast coil and a body coil. This metric is demonstrated to be tissue‐independent, allowing for easier SNR comparisons in cases of tissue deformation. We scanned 10 female subjects and compared the rSNR in segmented regions consisting of breast tissue, chest wall, and axilla between prone and supine breast imaging.

**Results:**

The rSNR was significantly higher in the breast tissue and chest wall in the supine position for all cases. The axilla rSNR was significantly higher in supine for four cases, with another four significantly higher in prone, and two showing no statistical difference. Using a distance‐from‐coil analysis, we found that the tissue is closer to the coil in supine, and that the supine coil provided higher SNR at distances closer than 4cm.

**Conclusion:**

Our results show that using a surface array coil in the supine position can provide higher SNR than a standard setup in most subjects for most relevant regions of breast MRI.

## 1 Introduction

Breast MRI has emerged as a key component to breast cancer screening, especially among women with a higher‐than‐average risk [1, 2, 3]. Additionally, recent studies support expanding MRI screening to women with an average risk for breast cancer, as well as those with dense breast tissue [4, 5, 6]. Recent developments in breast MRI include abbreviated breast MRI to reduce exam times [7], as well as diffusion‐weighted imaging to replace contrast injections [8, 9, 10, 11].

One less frequently considered aspect of breast MRI is patient positioning. Typically, breast MRI is performed in the prone position to mitigate respiratory and, to a lesser extent, cardiac motion artifacts. However, co‐localization of these images with ultrasound‐guided biopsy, lumpectomy, and whole breast irradiation, which are performed in the supine position, is difficult, due to the significant tissue deformations between the two positions [12, 13, 14]. Additionally, the prone setup tends to be exceedingly uncomfortable for patients, especially with the long exam time of the current protocols. This can lead to the patient moving during the scan, leading to motion artifacts and adjustments in position [15].

Recent efforts have focused on developing supine breast MRI, specifically to improve position alignment between MRI and surgical procedures [14]. Studies have shown the ability to perform both contrast‐enhanced MRI and diffusion MRI in the supine position [14, 16]. Additionally, to increase SNR and parallel imaging acceleration and accommodate different patient sizes, flexible coils have been developed specifically for supine breast MRI [17, 18].

Quantitatively evaluating potential differences between prone and supine positions is difficult due to the large tissue deformations and displacement between the positions. Comparisons usually require manual segmentation of an easily identifiable area that can be seen in both positions [14, 16]. Additionally, the MR signal in breast tissue is heterogeneous, primarily due to differing amounts of fibroglandular tissue and fat. It is also impractical to directly compare supine to prone imaging using the same coil, because standard coils cannot be used supine, and flexible coils provide no support for prone imaging. Ideally, we need a tissue‐independent metric that isolates the effect of patient positioning and coils on the MR signal.

We propose using relative SNR (rSNR) as a metric for tissue‐independent SNR comparisons between prone and supine breast coils. We then use this metric to quantitatively compare the impact on SNR between prone and supine breast MRI in several important regions. Additionally, the distance of the tissue from the nearest coil element was calculated to assess how it impacted SNR measurements. Initial versions of this work were presented at the 2024 and 2025 ISMRM annual meetings [19, 20].

## 2 Methods

We propose using relative SNR (**rSNR**) as a tissue‐independent metric. Relative SNR has been used previously to compare coil performance [21, 22], but its use as a tissue‐independent metric is novel. For clarity, rSNR is defined as follows: $rSNR=\frac{SNR_{ext}}{SNR_{ref}}$. $SNR_{ext}$ refers to the SNR of the primary coil used, whereas $SNR_{ref}$ refers to the SNR of the reference coil kept constant between the positions. Since the reference coil needs to be identical, the simplest choice is the transmit/receive (T/R) body coil in the MR scanner. rSNR enables tissue‐independent SNR comparisons that are particularly useful in breast tissue, as the SNR otherwise varies substantially between fibroglandular tissue and fat, requiring challenging segmentation for comparison of these tissue types.

### 2.1 Relative SNR Maps

To compute SNR maps, low‐resolution 3D‐Cartesian T1‐weighted gradient echo (SPGR) images were acquired, with frequency direction A/P and phase directions R/L and S/I. Coil sensitivity maps were estimated from the center 24×24×12 region of k‐space (windowed to reduce ringing). Images were reconstructed using standard unaccelerated SENSE reconstruction [23] using an R=1 SENSE constructor, as well as 2×4 accelerated SENSE (2× superior/inferior, 4× left/right) performed by retrospectively subsampling the unaccelerated data. Reconstructions were performed in MATLAB 2020b using custom reconstruction software. SNR maps were computed using the appropriate SENSE combination weights and the measured covariance matrix to obtain the noise in each voxel [23], then dividing the low‐resolution reconstructed image by the noise. Noise covariance matrices were calculated from pre‐scan data, which acquires a 2048‐sample noise block for the estimation.

### 2.2 rSNR Tissue Independence

Scans were performed on a 3T Signa Premier system (GE Healthcare) using a small 20‐channel anteriorly placed AIR^TM^ coil (GE Healthcare) and the T/R body coil embedded in the MR scanner.

To validate tissue‐independence, rSNR was compared in a phantom using T1‐weighted and T2‐weighted contrasts. A diffusion breast phantom (QalibreMD, Boulder, CO) was placed face up with the AIR coil draped over it and was scanned using 3D T1‐weighted SPGR and 3D T2‐weighted CUBE sequences. The phantom was scanned twice for each sequence, first using the AIR coil and then the T/R body coil as the reference. The 3D T1‐weighted SPGR sequence was acquired with the following scan parameters: $13^{\circ }$ flip angle, 2.1 ms TE, and 3.4 ms TR. The 3D T2‐weighted CUBE sequence was acquired with the following scan parameters: 90° flip angle, 83.5 ms TE, 2500 ms TR. Both sequences were acquired at 36 × 36 cm FOV, with 128 × 128 matrix, 66 slices, and 3 mm slice thickness.

### 2.3 Supine Vs. Prone rSNR

Seven patients with known breast lesions and three healthy volunteers (all F, aged 25–75) were recruited for a research breast MRI exam. The subjects had measured breast tissue volume ranging from 750–3000 mL. Following an IRB‐approved protocol, including written informed consent, subjects were scanned on a 3T Signa Premier System (GE HealthCare) in both supine and prone. The supine acquisitions used a 60‐channel flexible breast AIR coil [17] (GE Healthcare), while prone acquisitions used a 16‐channel Sentinelle Breast coil (In Vivo Corp, Gainsville, FL). The volunteers were additionally scanned supine with a 20‐channel AIR^TM^ coil.

In each position, a moderate‐resolution 3D T1‐weighted SPGR sequence was acquired using both the breast coil and T/R body coil. The 3D T1‐weighted SPGR sequence was acquired with the following scan parameters: flip angle 13°, 2.1 ms TE, 3.5 ms TR, total rBW 83 kHz. The FOV and matrix size were optimized for each position due to changing geometries, but kept the same for each pair of breast coil and T/R body coil acquisitions, so any voxel size changes would not impact rSNR calculations.

For each subject, prone and supine images were manually segmented into breast tissue, chest wall, and axilla regions (Figure 2). For segmentation, the breast tissue includes the skin, and the chest wall was defined as the region around the pectoralis major and pectoralis minor. The axilla was defined as the 3–4 cm diameter region around the axillary lymph nodes, posterior to the breast tissue and to the exterior of the chest wall. Segmentations were performed over all slices containing breast tissue by the same reader and were verified by a subspecialty‐trained Radiology resident with 3 years of breast imaging experience. An erosion analysis was also performed on the segmentations to verify reliability (Figure S2).

The rSNR measurements for all pixels over the entire segmented volume for each subject in both positions were compared statistically using a two‐tailed bootstrapped hypothesis test [24] for the difference between percentiles. The percentiles compared were the 5th, 25th, 50th, 75th, and 95th, and the significance level used was p<0.01.

rSNR was then used to investigate the performance of the prone and supine coils for different breast sizes. First, the median rSNR measurements were compared against subject breast volume, which was estimated using the segmented breast tissue. Finally, to assess the impact of positioning on coil‐to‐tissue proximity, the 3D distance from each voxel to the nearest coil element was calculated for each subject, grouped by the segmented tissue regions, as well as the median rSNR at each distance for both coils, binned into 2 mm chunks. The distances were calculated by estimating the location of the prone paddles in each subject as bars directly adjacent to the left and right sides of either breast. Since the supine coil is a flexible array coil, the distances in supine were estimated assuming the coil elements lay insulated against the skin.

## 3 Results

### 3.1 rSNR Tissue Independence

**FIGURE 1:**
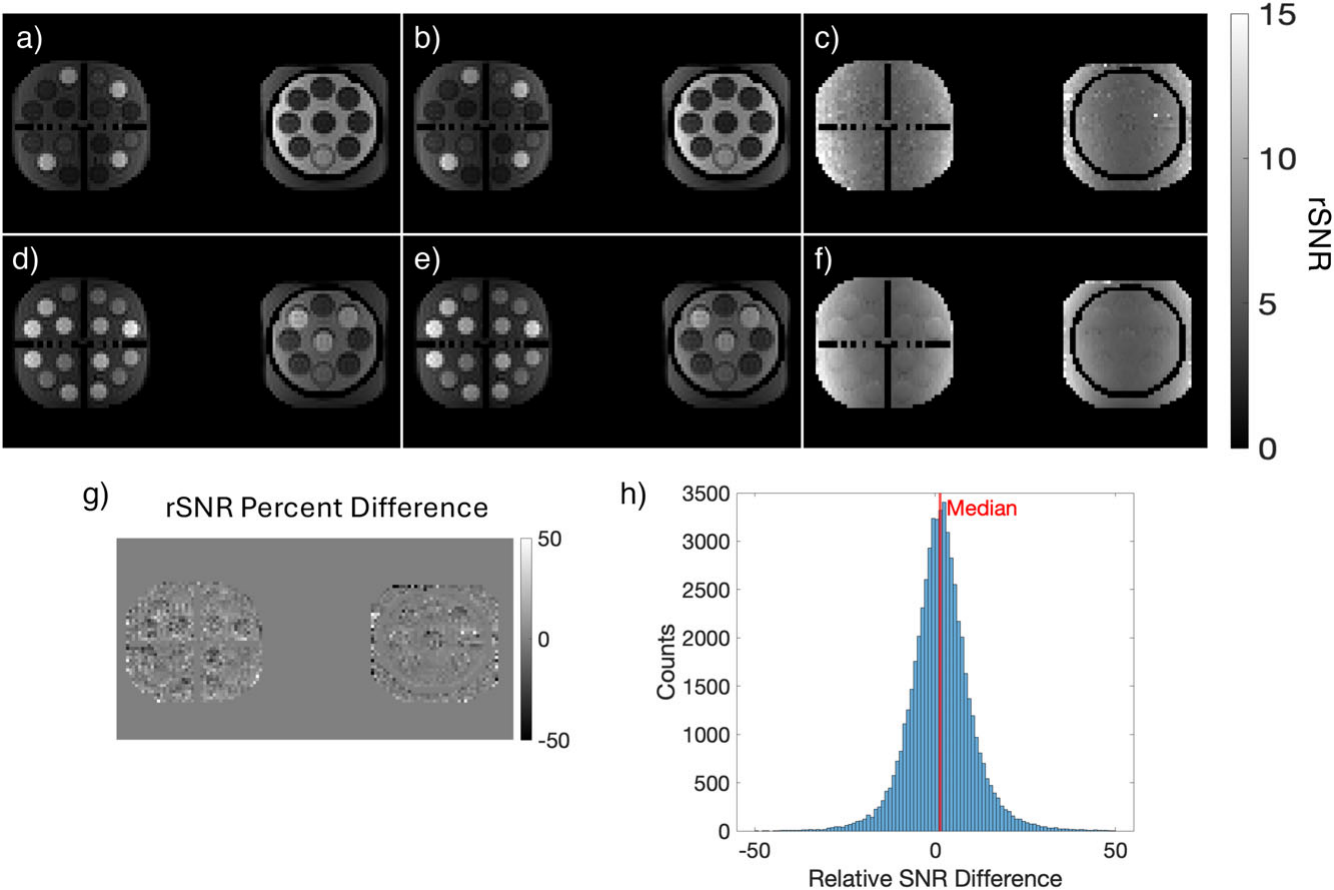
An example slice of the (a) T1‐ and (d) T2‐weighted images of the phantom are shown. The SNR maps for the same slice are also shown for the (b) T1‐and (e) T2‐weighted images. Relative SNR (rSNR) maps for the (c) T1‐ and (f) T2‐weighted images show a removal of the image contrast present in the raw images and SNR maps, albeit with some shading due to coil sensitivity variation across the image. An example slice of the (g) rSNR difference map, along with a (h) histogram of the differences across the entire volume, illustrates that the rSNR maps from either sequence are the same. The FWHM of the histogram is approximately 13.

The resulting T1‐weighted and T2‐weighted images, together with their rSNR maps, are shown in Figure 1. The T1‐ and T2‐weighted images show an obvious difference in contrast, with the vials clearly identifiable. The rSNR maps between the two datasets appear visually similar, and contrast from the phantom is removed, indicating a lack of signal dependence. A rSNR percent difference map (relative difference between rSNR for T1 and T2 images) was calculated for the whole volume and is shown in Figure 1 alongside a histogram of the differences. The difference map shows minimal structure, like the rSNR maps, and the distribution of the differences is centered close to 0, indicating the maps approach the same value. The FWHM of the histogram is approximately 13.

### 3.2 Supine Vs. Prone rSNR

**FIGURE 2:**
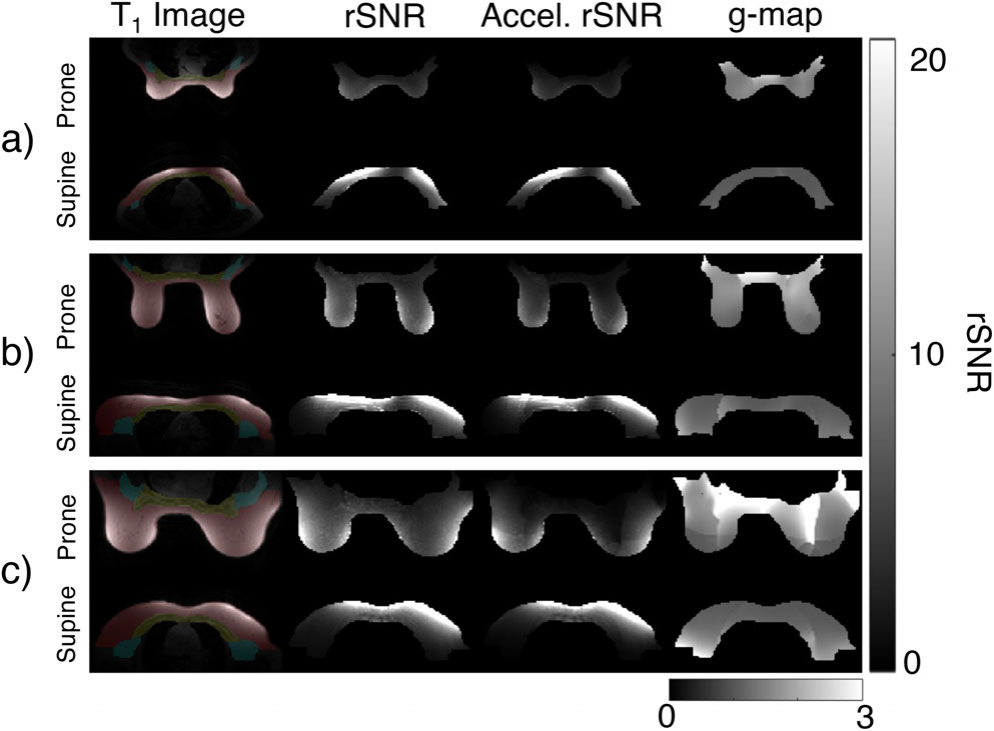
Anatomical and rSNR maps (with and without acceleration) for a central axial slice for (a) subject 4, (b) subject 6, and (c) subject 10 are shown, along with the g‐factor maps for the given acceleration. The segmentations for each subject are shown overlaid on the T1 image, with red indicating breast tissue, yellow indicating chest wall, and cyan indicating axilla. The subjects chosen varied in both breast size and shape, highlighting the possible patient variation in the clinic, as well as how that can affect rSNR.

Example slices of the anatomical T1‐weighted images, rSNR maps (with and without acceleration) and g‐factor maps are shown in Figure 2 for three cases. The segmentations for each subject are shown overlaid on the T1‐weighted images. The erosion analysis demonstrating the reliability of the segmentations for an example case is shown in Figure S2.

**FIGURE 3:**
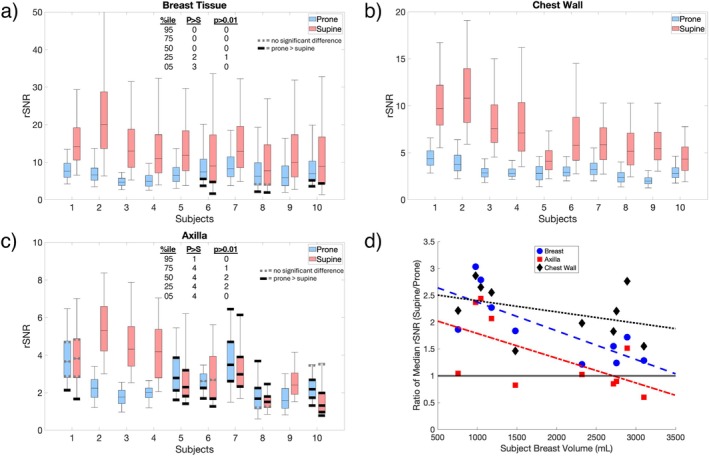
Box and whisker plots of rSNR distribution without acceleration in all subjects are shown for (a) breast tissue, (b) chest wall, and (c) axilla, ordered by subject breast volume from least to most. The top and bottom edges of the boxes represent the 75th and 25th percentiles, respectively, with whiskers extending out to the 5th and 95th percentiles. In the majority of cases and percentiles, the supine positioning and coil led to significantly higher relSNR. Tests where prone was significantly higher (“P>S”), or where the difference was not significant (p>0.01) are highlighted and totaled. Additionally, (d) the ratio of median rSNR in supine to prone as a function of the segmented subject breast tissue volume is shown for all three segmented regions. Trend lines, calculated using a linear regression fit, for each segmented region are also displayed. A horizontal line is also drawn at y=1 to indicate which position performs better for each point.

Volumetric distributions of unaccelerated rSNR measurements in all subjects are shown in Figure 3 for breast tissue, chest wall, and axilla, including median and 5/25/75/95th percentiles. In all subjects, the median breast tissue and chest wall rSNR was significantly higher (p<0.01) in supine. For 4 out of the 10 subjects, the axilla rSNR was significantly higher (p<0.01) in supine, with 4 others significantly higher (p<0.01) in prone. The remaining two subjects did not show a statistical difference (p<0.01) in the median rSNR between positions. Similar comparisons among the three coils for the three volunteers are shown in Figure S3, with the AIR coil rSNR being significantly higher (p<0.01) for two of the three volunteers.

The ratio of median rSNR in supine to prone for all subjects is plotted as a function of estimated subject breast tissue volume in Figure 3, again for breast tissue, chest wall and axilla. Calculations indicate that, for some subjects and tissue regions, the rSNR improvement in the supine position can be greater than a factor of 2. For the breast tissue and chest wall, the level of improvement tends to decrease with increasing breast volume. However, this trend is not seen in the axilla.

**FIGURE 4:**
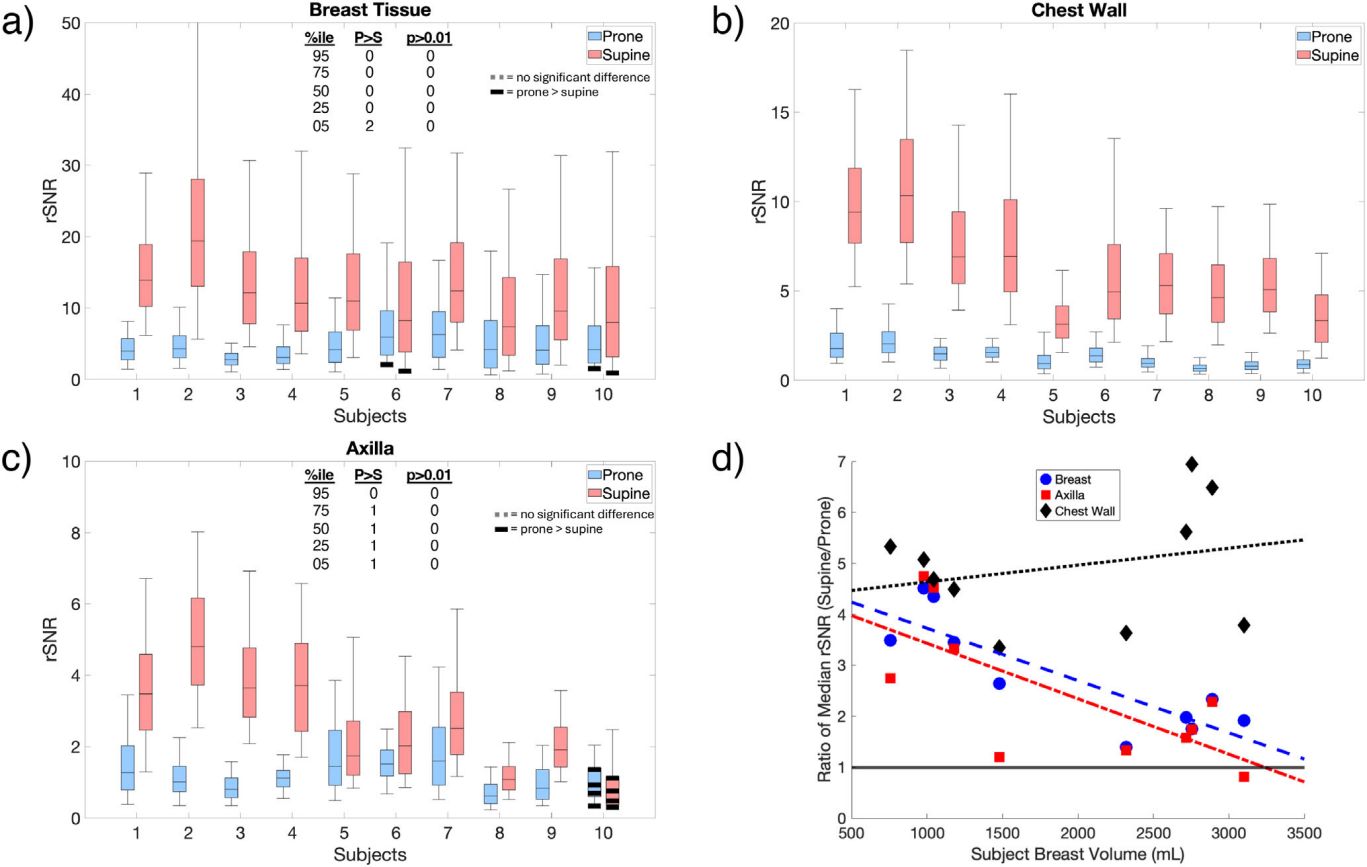
Box and whisker plots of rSNR distribution with 2×4 acceleration in all subjects shown for (a) breast tissue, (b) chest wall, and (c) axilla, ordered by subject breast volume from least to most. The top and bottom edges of the boxes represent the 75th and 25th percentiles, respectively, with whiskers extending out to the 5th and 95th percentiles. In the majority of cases and percentiles, the supine positioning and coil led to significantly higher rSNR. Tests where prone was significantly higher (“P>S”), or where the difference was not significant (p>0.01) are highlighted and totaled. Additionally, (d) the ratio of median rSNR in supine to prone as a function of the segmented subject breast tissue volume is shown for all three segmented regions. Trend lines, calculated using a linear regression fit, for each segmented region are also displayed. A horizontal line is also drawn at y=1 to indicate which position performs better for each point.

Similarly, volumetric distributions of the 2 × 4 accelerated rSNR measurements are shown in Figure 4. In all subjects, the median breast tissue and chest wall rSNR was significantly higher (p<0.01) in supine. For 9 out of the 10 subjects, the axilla rSNR was significantly higher (p<0.01) in supine, with only subject 10 significantly higher (p<0.01) in prone. The ratio of median rSNR in supine to prone for all subjects, plotted as a function of estimated subject breast tissue volume, is also shown in Figure 4. Compared to the unaccelerated case, the level of improvement increased for multiple subjects. The breast tissue and axilla trend lines show a steeper drop‐off in improvement as breast volume increases. Interestingly, the chest wall trend line shows a slight increase in improvement as breast volume increases, contrary to the unaccelerated case.

**FIGURE 5:**
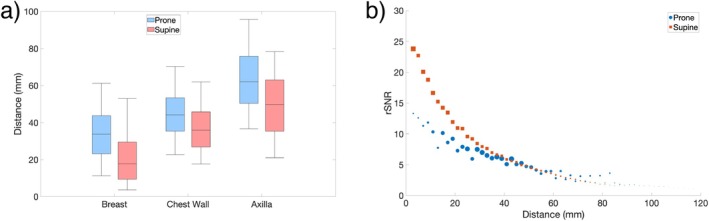
A box and whisker plot of the (a) distribution of pixel distances from the nearest coil element for all subjects is shown for breast tissue, chest wall, and the axilla. The top and bottom edges of the boxes represent the 75th and 25th percentiles, respectively, with whiskers extending out to the 5th and 95th percentiles. Additionally, the (b) median rSNR as a function of distance from the nearest coil element is also shown using values across all subjects, binned into 2mm chunks, with each marker size reflecting the relative amount of voxels at each distance. Notably, the median distance of pixels for a given sub‐region did vary between the two positions, with the coil elements closer in supine for all subjects in the breast and chest wall sub‐regions. Both coils had similar rSNR values at various distances from the coil elements, with the exception of tissue relatively close to the coil elements (< 40 mm).

Distributions of pixel distance from the nearest coil element are shown in Figure 5, alongside median rSNR as a function of distance from the coil for both positions. The median distance was lower in the supine position across all three segmented regions. For distances > 4 cm from the coil, both positions had similar rSNR values, but for distances < 4 cm, the supine setup had better rSNR values than the prone, increasingly so as the distance from the coil approaches 0. For the supine coil, the coil element diameter is roughly 7 cm [17], and such trends in SNR have been observed previously when increasing the number of smaller coil elements [25].

## 4 Discussion

The goal of this work was to compare SNR between supine and prone breast imaging, using a surface array coil and a standard breast coil, respectively. The tissue deformation between positions makes direct SNR comparison difficult, and the varying fat/water composition further complicates comparisons. rSNR provides a method to evaluate these SNR differences in patients using a basic segmentation of major tissue regions. Although the SNR calculation for this study used a basic SENSE reconstruction, other methods exist for more complex reconstructions [26, 27], and the rSNR framework can similarly be applied, including for different accelerations.

The phantom experiment demonstrates the signal independence of rSNR, which removes the contrast weighting in the resulting images. Example SNR maps of the T/R body coil (Figure S4) demonstrate that the T/R body coil is a reliable reference coil for rSNR calculations in practice. Additionally, any signal variation across the image is due to coil sensitivity variation across the image. The difference map shows similar rSNR maps derived independently from both sequences, with the distribution of differences centered near 0. The variation in pixel‐wise rSNR measurements between the two phantom scans is primarily caused by the acquired images having innate measurement noise (Figure S1). Specifically, in the calculations, the signal aspect of the SNR calculations inherently has noise associated with it, due to measurement noise of the scanner, and this noise propagates through the pixel‐wise rSNR calculations. However, this noise becomes much less of an issue when comparing large groups of voxels or analyzing overall trends.

In this study, breast tissue rSNR is higher with supine positioning over prone, with the chest wall showing similar improvement. This supports the reasoning that the breast deformation in supine allows for coil elements to sit closer to more of the tissue. This is further supported by the estimated distance from the nearest coil elements, with the supine positioning showing coil elements closer to both breast tissue and the chest wall for all subjects.

In breast MRI, breast size and shape can vary significantly between subjects, causing variations in image quality. This is exemplified by the variation in the axilla results, with some subjects showing better image quality in prone and others in supine. This variability seems to be driven by how the breast deforms in the supine position for subjects with larger breast size. Notably, in large breast size subjects, the breast tissue tends to fall away from the sternum, causing breast tissue to collect and cover the axilla. The result is reduced axilla SNR, which in some cases is lower than prone, where the axilla may be closer to the coil. For subject 6, this breast tissue deformation is severe enough to cause SNR to suffer in the breast tissue, with a substantial portion of the breast tissue falling outside the range of the coil elements. This could be mitigated in future supine coil designs that specifically account for how breast tissue deforms in subjects with larger breast sizes.

We observed varying levels of improvement between supine and prone imaging, depending on the subjects' breast size and in the different segmented regions. The chest wall showed the largest improvement between positions, with many subjects showing >2× factor increase, and all subjects showing >1.5× increase in rSNR. The factor of improvement in chest wall rSNR seemed to be related to subject breast size; specifically, subjects with smaller breast size generally had a larger improvement. Improvement in breast tissue rSNR also varied with subject breast size. Subjects with smaller breast sizes showed a level of improvement near or exceeding 2×, whereas subjects with larger breast sizes showed a level of improvement at or below 2×. The axilla was only slightly related to subject breast size, with improvements in axilla rSNR more pronounced in subjects with smaller breast sizes.

In a clinical setting, undersampling is frequently used to shorten exam times. In the case of 2 × 4 acceleration, we observed greater levels of improvement compared to the unaccelerated case. In the axilla, 9 subjects showed statistically higher median rSNR in supine, compared to only 4 subjects for the unaccelerated case. A major factor for this improvement is the g‐factor maps of the two coils, which are driven by a number of factors, including the number and density of coil elements. Additionally, factors like overall coil geometry and tissue proximity to coil elements also contribute to g‐factor improvement, illustrating the potential for supine positioning to additionally help under acceleration.

One major limitation of this work is the coupled nature of coil and position on the rSNR measurements. There are two variables that could be driving rSNR changes: coil or position. More specifically, both coils have different numbers of coil elements, which directly affects SNR. The distance‐from‐coil analysis was an attempt to partially separate these two variables and to determine which one was primarily driving the rSNR improvement. Ideally, with the same coil, the rSNR at each distance would be the same, and the only factor affecting rSNR would be the change in distance from the coil between positions. In reality, with the supine coil, we observe better rSNR closer to the coil than with the prone coil, while rSNR at > 4 cm is comparable to the prone coil. Since all segmented regions showed a closer distance to the coil in supine, it is likely that both positioning change and coil change positively impacted rSNR, but we are unable to determine the precise impact of each.

Another limitation of this work is the small sample size of only 10 subjects. A next step would be to replicate the work with a much larger sample size with a more diverse group of breast and patient sizes, since breast size and shape are highly variable in a clinical setting.

With supine positioning having higher rSNR, higher resolution breast MRI would become more feasible. Provided the coil provides sufficient SNR, any additional SNR could be used to improve the resolution of the scan, potentially allowing for better diagnosis. Resolution features in breast MRI are critical for accurate diagnosis, so enabling higher resolution imaging with a simple patient positioning change could impact diagnosis.

## 5 Conclusion

This study shows that rSNR can be a valuable tissue‐independent image quality metric to assess coil performance when tissue deforms. In the case of supine versus prone breast MRI, there was an improvement in rSNR when scanned supine for both breast tissue and the chest wall. The level of improvement of rSNR varied with subject breast size, with smaller breast sizes yielding larger factors of improvement. Overall, though there is still room for improvement in supine‐specific coil design, this study demonstrates the potential of supine breast MRI in improving SNR.

## Funding

This work was supported by GE Healthcare and the National Institutes of Health (Grant Nos. NIH R01‐EB009055 and R01‐CA249893).

## Conflicts of Interest

The authors receive research support from GE Healthcare.

## Supporting information




**Data S1**: **Figure S1:** A noise‐added simulation of rSNR measurements using Shepp‐Logan phantoms with two different signal profiles, (a) and (b). The baseline rSNR percent difference is shown in (c) and (d) for noise values similar to the phantom experiment, with the FWHM at 12.8. When decreasing the noise variance in both coils by a factor of 10, as in (e) and (f), we see much less variation, with the FWHM at 3.3. However, if we just decrease the noise variance in the external coil by 10 and leave the body coil noise the same, as shown in (g) and (h), we get a similar variation as in (c) and (d), with the FWHM at 11.2. This demonstrates both that the innate noise causes the variation, as well as how the variation depends on the noise level, specifically that it is dominated by the level of the largest noise (either body coil or external coil).
**Figure S2:** Erosion analysis of the segmentations was performed for one randomly selected subject. Each segmentation region was eroded at two different levels to simulate reader error in defining the region boundaries. The regions were eroded by either a 1‐pixel radius disk or a 2‐pixel radius disk. The results are shown in box and whisker plots of rSNR for (a) breast tissue, (b) chest wall, and (c) axilla. The top and bottom edges of the boxes represent the 75th and 25th percentiles, respectively, with whiskers extending out to the 5th and 95th percentiles. As can be seen, the general rSNR distributions remain relatively stable despite the erosion, pointing to the fact that the segmentations are good enough for the purposes of this study.
**Figure S3:** Box and whisker plots of rSNR distribution in the volunteers for the prone coil, supine‐specific coil, and a generic AIR coil in supine are shown for (a) breast tissue, (b) chest wall, and (c) axilla, ordered by subject breast volume from least to most. The top and bottom edges of the boxes represent the 75th and 25th percentiles, respectively, with whiskers extending out to the 5th and 95th percentiles. As can be seen, the supine‐specific coil generally performs the best, with the prone coil generally performing the worst, and the air coil performing somewhere in between. This reinforces the hypothesis that supine positioning is a factor in the rSNR gain seen, and it's not exclusively a coil‐specific gain. Note that the AIR coil has only 20 coil elements compared to the supine‐specific coil's 60 coil elements, so the number of coil elements there is more comparable to the prone coil, which has 16 coil elements.
**Figure S4:** Example slices of raw SNR T/R body coil maps are shown for two randomly selected subjects in both prone and supine. As can be seen, the SNR values are close, reinforcing the stated assumption of using the T/R body coil as an appropriate reference.

## Data Availability

The data that support the findings of this study are available from the corresponding author upon reasonable request.
