# Supplementary material for: Relative SNR Measurements in Supine vs. Prone Breast MRI
Source: Magn Reson Med. 2025 Dec 25;95(5):2718–25. doi: 10.1002/mrm.70217 (PMC12962210; doi:10.1002/mrm.70217)
Supplement: Supplementary file 1 — Data S1: Figure S1: A noise‐added simulation of rSNR measurements using Shepp‐Logan phantoms with two different signal profiles, (a) and (b). The baseline rSNR percent difference is shown in (c) and (d) for noise values similar to the phantom experiment, with the FWHM at 12.8. When decreasing the noise variance in both coils by a factor of 10, as in (e) and (f), we see much less variation, with the FWHM at 3.3. However, if we just decrease the noise variance in the external coil by 10 and leave the body coil noise the same, as shown in (g) and (h), we get a similar variation as in (c) and (d), with the FWHM at 11.2. This demonstrates both that the innate noise causes the variation, as well as how the variation depends on the noise level, specifically that it is dominated by the level of the largest noise (either body coil or external coil). Figure S2: Erosion analysis of the segmentations was performed for one randomly selected subject. Each segmentation region was eroded at two different levels to simulate reader error in defining the region boundaries. The regions were eroded by either a 1‐pixel radius disk or a 2‐pixel radius disk. The results are shown in box and whisker plots of rSNR for (a) breast tissue, (b) chest wall, and (c) axilla. The top and bottom edges of the boxes represent the 75th and 25th percentiles, respectively, with whiskers extending out to the 5th and 95th percentiles. As can be seen, the general rSNR distributions remain relatively stable despite the erosion, pointing to the fact that the segmentations are good enough for the purposes of this study. Figure S3: Box and whisker plots of rSNR distribution in the volunteers for the prone coil, supine‐specific coil, and a generic AIR coil in supine are shown for (a) breast tissue, (b) chest wall, and (c) axilla, ordered by subject breast volume from least to most. The top and bottom edges of the boxes represent the 75th and 25th percentiles, respectively, with whiskers extending out to the 5th a [file MRM-95-2718-s001.docx]

**Supporting Information**

**Supporting Information Figure S1:** A noise-added simulation of rSNR measurements using Shepp-Logan phantoms with two different signal profiles a) and b). The baseline rSNR percent difference is shown in c) and d) for noise values similar to the phantom experiment, with the FWHM at 12.8. When decreasing the noise variance in both coils by a factor of 10 as in e) and f), we see much less variation, with the FWHM at 3.3. However, if we just decrease the noise variance in the external coil by 10 and leave the body coil noise the same, as shown in g) and h), we get a similar variation as in c) and d), with the FWHM at 11.2. This demonstrates both that the innate noise causes the variation, as well as how the variation depends on the noise level, specifically that it is dominated by the level of the largest noise (either body coil or external coil).

**Supporting Information Figure S2:** Erosion analysis of the segmentations was performed for one randomly selected subject. Each segmentation region was eroded at two different levels to simulate reader error in defining the region boundaries. The regions were eroded by either a 1-pixel radius disk or a 2-pixel radius disk. The results are shown in box and whisker plots of rSNR for a) breast tissue, b) chest wall, and c) axilla. The top and bottom edges of the boxes represent the 75th and 25th percentile respectively, with whiskers extending out to the 5th and 95th percentiles. As can be seen, the general rSNR distributions remain relatively stable despite the erosion, pointing to the fact that the segmentations are good enough for the purposes of this study.

**Supporting Information Figure S3:** Box and whisker plots of rSNR distribution in the volunteers for the prone coil, supine-specific coil, and a generic AIR coil in supine is shown for a) breast tissue, b) chest wall, and c) axilla, ordered by subject breast volume from least to most. The top and bottom edges of the boxes represent the 75th and 25th percentile respectively, with whiskers extending out to the 5th and 95th percentiles. As can be seen, the supine-specific coil generally performs the best, with the prone coil generally performing the worst, and the air coil performing somewhere in between. This reinforces the hypothesis that supine positioning is a factor in the rSNR gain seen, and it’s not exclusively a coil-specific gain. Note that the AIR coil has only 20 coil elements compared to supine-specific coil’s 60 coil elements, so the number of coil elements there is more comparable to the prone coil, which has 16 coil elements.

**Supporting Information Figure S4:** Example slices of raw SNR T/R body coil maps are shown for two randomly selected subjects in both prone and supine. As can be seen, the SNR values are close, reinforcing the stated assumption of using the T/R body coil as an appropriate reference.
